# Dolichoarteriopathy of Common Carotid Artery: An Unusual Entity

**DOI:** 10.1055/s-0042-1757951

**Published:** 2022-12-20

**Authors:** Efstratios Georgakarakos, Aliki Fiska

**Affiliations:** 1 Department of Vascular Surgery, “Democritus” University of Thrace, University Hospital of Alexandroupolis, Alexandroupolis, Greece; 2 Department of Anatomy, Medical School of Alexandroupolis, Democritus University of Thrace, Alexandroupolis, Greece

**Keywords:** common carotid artery, dolichoarteriopathy, tortuosity, congenital anomalies, kinking, coiling, elongation

## Abstract

Dolichoarterial disease of the carotid arteries refers to elongated arteries with tortuous, coiling, and kinking anatomy. This morphology is usually met in the elderly and not associated with atherosclerotic risk factors. Current practice reserves surgical correction only in symptomatic patients. Significant tortuosity index may be associated with stroke and poses extra difficulties to the endovascular passage of guidewires and catheters for the treatment of extra- and intracranial vascular lesions. This article presents a typical case of bilateral dolichoarteriopathy of the common carotid artery and stresses the need for further categorization of the particular morphology based on modern angiography techniques and three-dimensional reconstruction software.

Dolichoarterial disease (DAD) refers to elongated arteries with tortuous, coiling, and kinking anatomy. 1 Dolichoarteriopathies of carotid arteries are frequent, ranging between 10 and 45%. The incidence rate increases with increasing age and is high in the elderly, particularly in patients older than 70 years. Congenital anomalies such as fibromuscular dysplasia might also genetically predispose patients to DAD. This anatomical entity may lead to turbulent flow favoring thrombus formation and predisposing to cerebral embolism (transient ischemic attack or stroke). Hemodynamically, different degrees of angulation or kinking have been associated with different degrees of flow reduction and consequent hemodynamic cerebral changes or ocular vascular insufficiency. Furthermore, transient hypotension, such as when occurring during sleep, upon neck extension/bending, or when turning the head from side to side, can make the kinked artery collapse at the point of maximal angulation and reduce the blood flow causing transient cerebral ischemia. Finally, DAD may predispose to dissection.

Current guidelines of the European Society for Vascular Surgery suggest surgical correction only in symptomatic patients with isolated coils/kinks, provided that all other causes for transient ischemic attack or stroke have been excluded by multidisciplinary team review. 2 In general, the surgical approach focuses on the resection of the abundant vessel part, if surgically accessible, and end-to-end anastomosis or bypass grafting.

Bilateral dolichoarteriopathy ( Fig. 1 ) of the common carotid arteries is rare and does not seem to correlate with atherosclerotic risk factors. 3 Yet, the tortuosity index (i.e., the ratio of the length of a vessel segment to the distance between its start and endpoint) rather than the length of the carotid arteries may be associated with stroke. Moreover, these anatomical patterns pose extra difficulties to the endovascular passage of guidewires and catheters for the treatment of extra- and intracranial vascular lesions. 4 Three-dimensional reconstruction techniques can help to further categorize and assess relevant risks associated with endovascular procedures. Computed tomography angiography or magnetic resonance angiography assesses the morphology of the lesion. Yet, Doppler ultrasonography can assess flow changes in dynamic conditions or reproduce symptoms, such as with neck extension or bending or when turning the head from side to side.

**Fig. 1:**
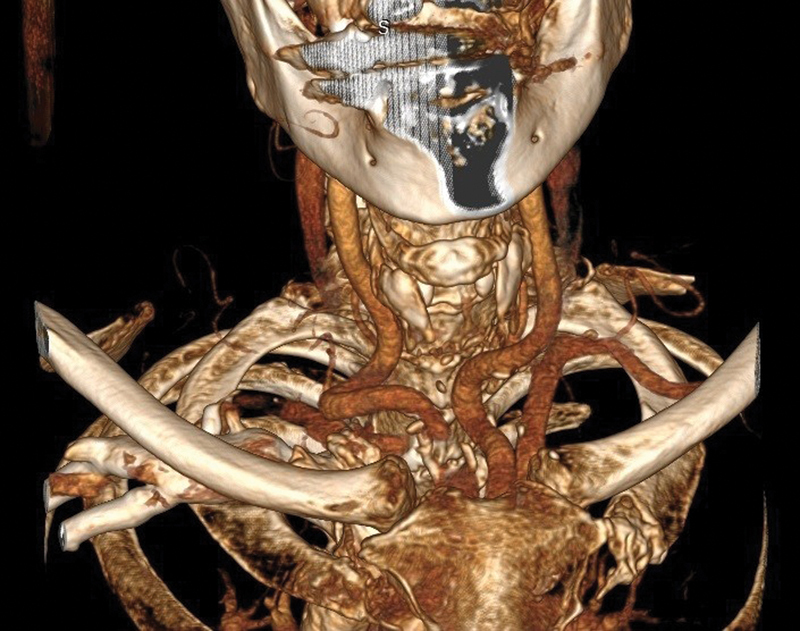
Bilateral dolichoarteriopathy of the common carotid arteries.
